# Bioinspired Designs, Molecular Premise and Tools for Evaluating the Ecological Importance of Antimicrobial Peptides

**DOI:** 10.3390/ph11030068

**Published:** 2018-07-10

**Authors:** Elvis Legala Ongey, Stephan Pflugmacher, Peter Neubauer

**Affiliations:** 1 Department of Biotechnology, Technische Universität Berlin, Ackerstraße 76, ACK24, D-13355 Berlin, Germany; peter.neubauer@tu-berlin.de; 2 Aquatic Ecotoxicology in an Urban Environment, Ecosystems and Environment Research Program, Faculty of Biological and Environmental Sciences, University of Helsinki, Niemenkatu 73, FI-15140 Lahti 2, 00100 Helsinki, Finland; stephan.pflugmacher@helsinki.fi; 3 Korean Institute of Science & Technology Europe, Joint Laboratory of Applied Ecotoxicology, Campus E7 1, 66123 Saarbrücken, Germany

**Keywords:** antimicrobial peptides, ecotoxicity, bacteriocins, therapeutic, polyproline helix, membrane disruption

## Abstract

This review article provides an overview of recent developments in antimicrobial peptides (AMPs), summarizing structural diversity, potential new applications, activity targets and microbial killing responses in general. The use of artificial and natural AMPs as templates for rational design of peptidomimetics are also discussed and some strategies are put forward to curtail cytotoxic effects against eukaryotic cells. Considering the heat-resistant nature, chemical and proteolytic stability of AMPs, we attempt to summarize their molecular targets, examine how these macromolecules may contribute to potential environmental risks vis-à-vis the activities of the peptides. We further point out the evolutional characteristics of the macromolecules and indicate how they can be useful in designing target-specific peptides. Methods are suggested that may help to assess toxic mechanisms of AMPs and possible solutions are discussed to promote the development and application of AMPs in medicine. Even if there is wide exposure to the environment like in the hospital settings, AMPs may instead contribute to prevent healthcare-associated infections so long as ecotoxicological aspects are considered.

## 1. Introduction

**Figure 1:**
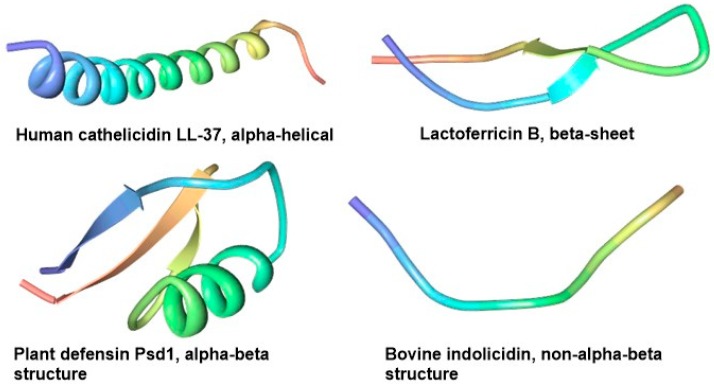
3D structures of selected AMPs, depicting four structural families based secondary structures found in the compound: alpha, beta, alphabeta and non-alphabeta. Structures were edited with protein workshop using the solution NMR structural data for Human cathelicidin LL-37 (PDB: 2K6O), lactoferricin B (PDB: 1LFC), plant defensin Psd1 (PDB: 1JKZ) and bovine indolicidin (PDB: 1G89).

There are many classes of natural compounds, five of which are prevalent in natural product research today, namely: polyketides, alkaloids, terpenoids, ribosomal and non-ribosomal peptides [1], the latter two groups are called antimicrobial peptides (AMPs). AMPs, also known as host defense peptides evolved from ancient times and are an integral component of the innate immune system of most organisms where they function as effector molecules. AMPs may be classified based their biosynthesis mechanism (ribosomal and non-ribosomal), biological sources (bacterial, plants, animal etc.), biological functions (antibacterial, antiviral, antifungal etc.), peptide properties (hydrophobic, amphipathic, cationic etc.), covalent bonding pattern (disulfide bonds, N- to C-termini peptide linkage etc.), 3D structure (alpha-helical, beta-sheet, alpha-beta and non-alpha-beta) and molecular targets (cell surface, and intracellular targeting peptides). Figure 1 presents the 3D structures of some common AMPs from plant and animal sources. AMPs occur naturally in many forms most of which are cationic amphiphilic compounds, constituting evolutional host defense machineries present in different species of all life forms.

The fundamental differences that exist between single-cell and multicellular organisms allow them to be targeted differently by AMPs. Some exhibit activities against wide variety of targets such as viruses, bacteria and fungi [2,3,4]. Others may function beyond the margins of antimicrobials to include disruption of tumour-infected cells [5] as well as modulating organ activity like the cathelicidin AMP which regulates pancreatic islets [6]. Furthermore, rational modifications and the in vivo incorporation of unnatural amino acids into the peptides may further expand the chemical space and provide new diversified functional characteristics [7], including, but not limited to, modulation of chemotaxis, cytokine release and wound healing. Although these modifications may enhance the applicability of the desired compound, little attention is given to the possible effects of such stable molecules on the surrounding environment especially with regards to degradability and the prevention of tolerant bacteria from emerging.

Early studies generally considered the bacterial cytoplasmic membrane as a unique target for AMPs but within the last two decades several reports describing alternative mechanisms of action exist [8,9,10,11,12,13]. They function over wide pH ranges [14,15], showing extreme stabilities to heat and protease degradation [16,17,18], and very little or no development of microbial resistances. While these characteristics are generally regarded as attractive features of the peptides, only the latter seems more useful in terms of safety. The fact that AMPs are currently contemplated for potential pharmaceutical use as alternatives to conventional drugs, with a host of them already undergoing clinical trials [19], extensive investigations are required to determine safety of modified analogues and rationally designed synthetic members. AMPs may also be widely implemented in food processing [20]. Most AMPs are obtained from natural origins [21,22], which makes them generally considered as safe for human consumption as opposed to their synthetic counterparts. Interestingly, the market for peptide drugs is increasing steadily as well [23]. However, clinical development and eventually therapeutic application of AMPs are usually thwarted by high manufacturing costs, host toxicity and substandard pharmacokinetic properties which are primarily attributed to their susceptibility to proteolytic inactivation and sustained serum binding in physiological environments [24,25,26,27].

Several approaches have been proposed and are currently utilized to improve these challenges created by the intrinsic properties of AMPs [28,29,30,31]. In-depth knowledge of structure-activity relationships has enabled the design of synthetic AMP mimics with biocompatible properties, providing another valuable strategy which is also presently employed by many research groups to address some of the drawbacks. Results of these approaches have been reported in many examples like the cationic β-stranded peptide [32], non-membrane-lytic peptides [33], the α-helical OP-145 [34] and the amphipathic helical WR12 and β-sheet D-IK8 peptides [35], which all showed potent antibacterial activities and reduced toxicity to human cells. The quest for new anti-infective compounds is growing over time but information on how to evaluate the ecological effects of these molecules are relatively scarce. The fact that most (if not all) AMPs interact with the lipid bilayer membranes is not a matter to be taken lightly since virtually all organisms contain this component. A disturbance on the bilayer membranes may weaken their integrity and make them more susceptible destruction, such as lipid peroxidation. Moreover, some can even penetrate and interact with intracellular components and as such, artificial AMPs in particular must be evaluated for possible ecotoxicological effects. 

Remarkably, some AMPs have structural similarities to well-known toxins like the spider neurotoxin named Cm38 and Cn11, producing considerable toxicity to mammalian cells by themselves [36,37,38]. It is therefore important to tackle procedures aimed at improving therapeutic qualities of AMPs with caution as novel categories of toxins may evolve in the process. Furthermore, we think that the ability for an AMP to resist heat, protease and chemical inactivation may not only be considered with respect to the benefits those may offer, but also to observe that they may well be difficult to degrade when the need arises. Thus, their investigations must also seek to address some critical feasible queries, which are the possible inactivation/excretion routes and probable side effect if the peptide accumulates in body organs. Perhaps there may be potential ecological effects if the molecules are excreted in their full form. In the present review, we highlight structure-function relationships and mode of action of AMPs that may threaten environmental safety while discussing the use of artificial and/or natural AMPs as templates for rational design of peptidomimetics. We further propose some strategies to reduce cytotoxic effects of AMPs against eukaryotic cells. We also attempt to summarize different molecular targets identified so far, evaluate their possible contributions to environmental instability vis-à-vis the activities of peptides, and suggest methods that may help to assess their ecotoxic mechanisms. Our suggestions are supported by the facts that AMPs may leach via AMP-coated devices, manuring and aquaculture systems, to the surrounding environments; or the peptides may be consumed as drugs or in food (as preservatives) and subsequently excreted into aquatic environments from home reservoirs. However, AMPs are highly potent against pathogenic microbes, targeting multiple cellular macromolecular structures whose constituent building blocks or conformational dynamics vary from species to species and therefore, resistance development is rare. As such, the above phenomena may instead contribute to prevent healthcare-associated infections in hospital settings and food-borne illnesses so long as environmentally friendly species are not targeted.

## 2. Diverse Sources of AMPs

**Figure 2:**
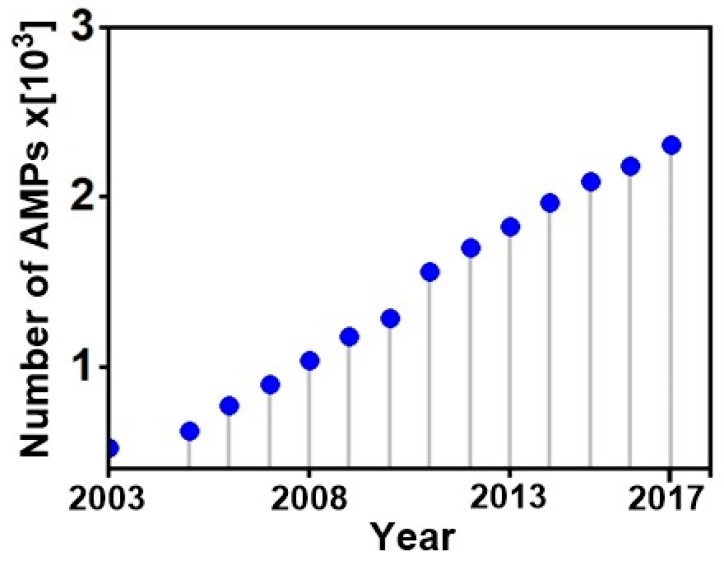
Number of AMPs isolated from natural sources since 2003 (information was extracted from the AMP database (http://aps.unmc.edu/AP).

Genome sequencing and transcriptomic data show that AMPs are ubiquitous in nature, and present in almost all living organisms [39,40,41,42,43], providing defensive roles that significantly protect the host from competing organisms in their respective ecological niches and ensure their survival [44]. Several different AMP families exist (e.g., bacteriocins, defensins etc.), that are isolated from various natural sources with bacterial [39,45], plant [46] and animal [47,48,49], hosting the majority; while a few are derived from archaea [50], protists [51] and fungi [52]. They may also be derived from chemical synthesis [53]. In the last years the number of newly identified AMPs increased rapidly, supported by the fact that the methods for the isolation, mass detection, sequencing and even structural elucidation have become standard technologies [54]. Wang et al. (2015) reported a total of 2493 registered members (including artificial ones) in the AMP database (APD) at the end of December 2014 [55], 122 natural peptides were isolated in 2015, 86 in 2016, 124 in 2017, adding to 17 new members already reported in 2018 making a total of 2981 compounds hosted by the APD, with well-established sequences and functions. Figure 2 shows a quasi-linear increase in the number of AMPs isolated from natural sources over the last 15 years.

### 2.1. Structural Diversity of AMPs from Non-Bacterial Sources

**Figure 3:**
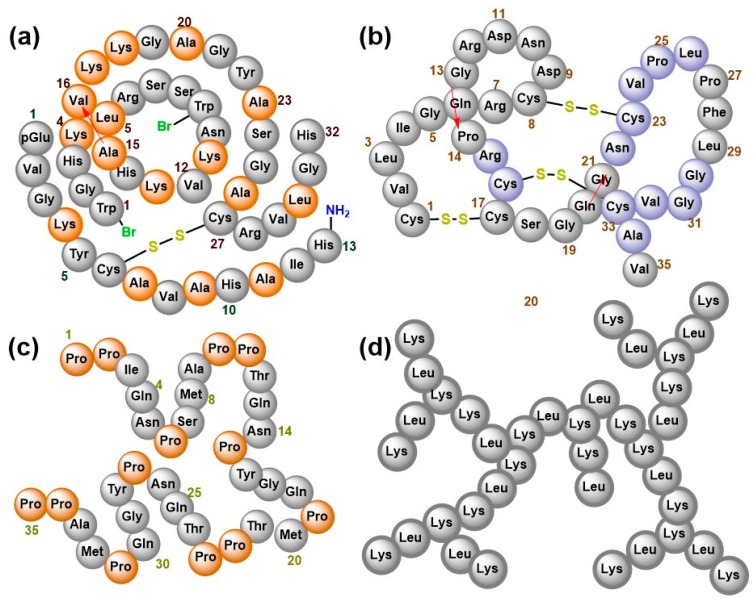
Diversified schematic structures of natural and artificial AMPs. (**a**) EeCentrocin 2 is an α-helical heterodimeric peptide with a heavy and a light chain connected via a disulfide bridge (helix-stabilizing residues are indicated in light brown). (**b**) EPrAMP1 has three disulfide bridges and three strands of beta-sheet (shown in violet). (**c**) BnPRP1 is a proline-rich peptide. (**d**) G3KL is a tree-like synthetic polymer composing of alternating residues of lysine and leucine. Red arrows indicate direction of crossing in the peptide chain.

AMPs from plants and animal sources have enormous structural diversity including those described earlier (Figure 1) [56], as well as those illustrated by the examples of EeCentrocin 2 from *Echinus esculentus* [57] and EPrAMP1 from *Echinopsis pachanoi* [37] (Figure 3a,b, respectively). α-helical peptides are usually between 12 and 40 amino acid long and are rich in residues that favour the α-helical conformation like Ala, Leu and Lys. They assume an amphipathic structure when interacting with the membrane [58]. They usually do not contain Cys, but some example like the 11- and 5-kDa peptides purified earlier from guinea pig neutrophils [59] and EeCentrocin 2 (Figure 3a) possesse a unique structural feature by forming an intermolecular disulfide between a heavy chain and a light chain [57]. An additional N-terminal pyroglutamic acid and a C-terminal amidation of the light chain of EeCentrocin 2, as well as the 6-Br-Trp modifications of the heavy chain do not affect its antibacterial function, indicating that these elements may be ascribed to the biostability of the peptide [57].

Contrary to the α-helical peptides, those that adopt the β-sheet conformations contain at least two Cys residues that form between 1 and 5 intramolecular disulfide bonds [60] that stabilize the tertiary structure of the peptides and confer substantial resistance to heat, protease and enzymatic inactivation [61,62]. β-hairpin structures are also common for those containing 2–4 Cys residues e.g., protegrin-1 [63]. A structural motif referred to as the inhibitor cystine knot is an evolutionary conserved feature shared amongst β-sheet peptides as well as the TGF-β/BMP family of regulatory cysteine knot proteins [64]. They provide the conformational correctness that allows the peptides to exhibit diverse biological roles such as antimicrobial, anti-HIV, insecticidal, protease inhibition and G-protein-coupled receptors regulatory activities [62,65,66,67,68]. The representative example of EPrAMP1 (Figure 3b) has three disulfide bridges involving (i) Cys1–Cys17, (ii) Cys8–Cys23 and (iii) Cys16–Cys33 disulfide bonds. The interconnecting backbones of (i) and (ii) constitute a ring that impregnates (iii), yielding a structural arrangement that stabilizes three strands of β-sheet covering residues 7–8, 22–26 and 30–34 [37]. The disulfide bonds and stabilized secondary structure contribute extensively to the overall tertiary folded structure which has a flat, disc-shaped appearance.

BnPRP1 from *Brassica napus* (Figure 3c) like many other proline-rich AMPs (PrAMPs) forms random coils with little amount of α-helix composition that engage in a non-lytic modes of bacterial inactivation [69]. Since proline is incompatible with α-helical or β-sheet conformation, its frequent occurrence in a polypeptide sequence causes a conformational rearrangement that forces the molecule to adopt the polyproline II (PPII) helical structure [70,71,72] and ample evidence suggest that the PPII conformation may be the biologically active form for all PrAMP [72,73,74,75,76,77,78,79].

The PP-II helix is an extended polypeptide structure which is arranged such that it consists of three amino acid residues per turn [80], and it is however, not the only component required to produce antimicrobial activity as Guzmán et al. (2013) showed, that the PPII-like secondary structure of proline homopeptides alone is not enough to elicit bacterial inhibitory effects. This demonstrated the distorting effects of the cyclic side chain of proline that forms a rigid conformation in PrAMPs which may constraint flexible movement at the membrane interface that is necessary to enable the peptide to adopt different structural forms while interacting with the membrane [81].

Studies show that synthetic peptides that are predominantly composed of Lys residues also fold into PPII helix in aqueous solution [72,78], and AMPs with such amino acids composition can interact with bacterial membranes and eventually penetrate into the cell [82,83]. Other studies also revealed that odd-numbered residues of Lysine homopeptides elicit full growth inhibition of Gram-positive and Gram-negative bacteria [72,78]. However, the PPII helix does not seem to apply to the biologically active dendritic topology of G3KL (Figure 3d) whose alternating residues of lysine and leucine produces tree-like branches that kill Gram-negative strains, exhibiting low toxicity to red blood cells [84].

Rationally designed AMPs like the G3KL, IK12 [85] and K4R2-Nal2-S1 [86], mostly target the cell membranes since the functional relationships with this component have been investigated extensively. Although the killing mechanisms of some of the newly identified peptides are not yet investigated, the fact that TLN-58 derived from eccrine sweat upregulates interleukins especially IL-8 [20] suggests that its mode of action may involve chemotaxis and peptide receptor–like induction of angiogenesis which may be used in therapeutic procedures involving mesenchymal stem cells for the treatment of diseases like stroke and spinal cord injury [28]. Similarly EeCentrocins are rich in hydrophobic and positively charged amino acids, hence they contain significant cationicity and hydrophobicity that are necessary to form an amphipathic structure that may enable the peptides to act on the membranes of Gram-positive bacteria because the hydrophilic portion enables interaction with the polar heads, while the hydrophobic regions interact with the hydrophobic core of the lipid bilayer membrane [57]. Summarily, majority of AMPs from sources other than bacteria kill mostly Gram positive bacteria except for a few like G3KL, Thaulin-1 from *Pleurodema thaul* [87], BnPRP1, *Mj*Pen-II from *Marsupenaeus japonicus* [15] and Scolopendin from *Scolopendra subspinipes mutilans* [88] which are able to kill Gram negative and/or other microorganisms. Moreover, some function beyond microbial inhibition/killing e.g., K4R2-Nal2-S1 may be used to treat cancer [86] and TLN-58 for immune modulation [20] as described above.

Summarily, we have seen that a wide variety of structural motifs exist in eukaryotic AMPs (and rationally designed peptides), but they possess an amphipathic structure that is a common feature to all of them. This feature enables them to bind and interact at the lipid bilayer membrane interface. The fact that most of these peptides interact with the lipid bilayer membranes is particularly concerning since such disturbance on the membranes may weaken their strength and render the cells more prone to lysis. Moreover, some AMPs (mostly from plants and insects) have the ability to penetrate the membrane and interact with intracellular processes and as such, this must be kept in mind when modifying or designing a new molecule to ensure its relevance and safety.

### 2.2. Structural Diversity of AMPs from Bacterial Sources

AMPs produced by bacteria are generally called ribosomal or non-ribosomal peptides based on their biosynthesis mechanism. Those that are ribosomally synthesized are generally referred to as bacteriocins. Bacteriocins are defined as “a group of proteinaceous substances produced at ribosomal level, having multifunctional properties whose antimicrobial activities are concentration-dependent [89]”. This definition largely eliminate the general consideration that they are amphipathic in nature and possessing an overall positive charge, because anionic AMPs such as subtilosin A do exist [90]. Bacteriocins are prevalent amongst all species of bacteria especially the lactic acid bacteria (LAB). They are grouped into three main classes namely; post-translationally modified bacteriocins (class I), unmodified bacteriocins (class II) and bacteriolysin (class III) [91]. Bacteriocins usually inactivate their targets via membrane disruption or by forming pores, or even terminate cell division by targeting and dissociating lipid II which serves as a precursor molecule during bacterial cell wall synthesis [91].

**Figure 4:**
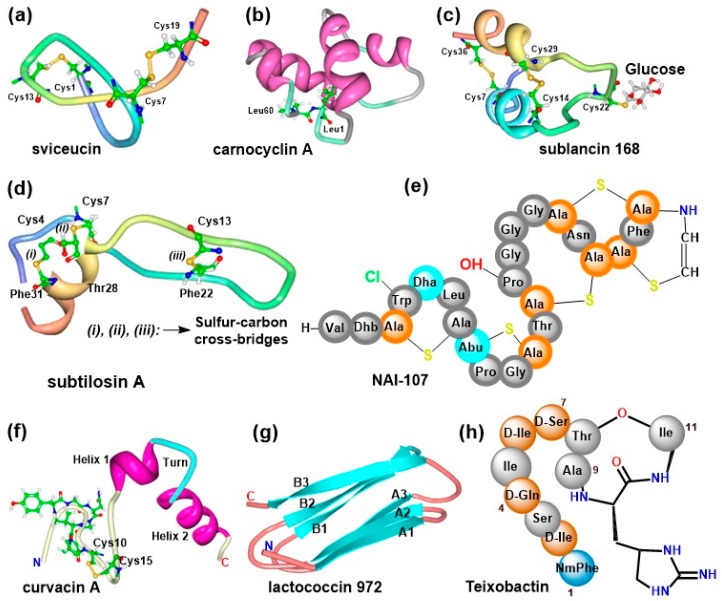
Structure of selected peptides from bacterial sources. (**a**) Lasso peptides sviceucin, showing two intramolecular disulfide bridges. (**b**) Circular peptide carnocyclin A, showing N- to C-terminal peptidyl linkage between Leu1 and Leu60. (**c**) Glycocin peptide sublancin 168, showing a glucose moiety linked to Cys22 and two intramolecular disulfide bridges. (**d**) Sactipeptides subtilosin A, showing the coordination of the sulfur-α-carbon bridges. (**e**) NAI-107 has 5 intramolecular thioether cross-linkages, additional 5-chloro-trypthopan, mono-/bis-hydroxylated proline and a c-terminal aminovinylcysteine. (**f**) Pediocin-like curvacin A, showing the N-terminal YGNGVXC conserved motif, the N-terminal intramolecular disulfide bridge between to Cys10 and Cys15, and the distinct C-terminal helical region. (**g**) Non-pediocin-like single-peptide lactococcin 972. (**h**) Non-ribosomally synthesized peptide teixobactin compose of four d-amino acids, enduracididine and an N-terminal methylphenylalanine. Structures were edited with protein workshop using the solution NMR structural data for each molecule.

Research has demonstrated that amongst the different families of AMPs, bacteriocins may be used as alternatives to conventional antibiotics based on their diverse structural characteristics and remarkable potencies against drug-resistant bacteria strains [45]. Class I bacteriocins are more interesting with respect to the diverse structural forms present in this group. A subclass referred to as lasso peptides possess a characteristic ring formed via an amide bond between the first residue of the core peptide and a negatively charged core residue at positions +7, +8 or +9; after which the ring then embodies the linear C-terminus of the sequence [1,91,92]. Additional modifications such as intramolecular disulfide bridges like in the case of sviceucin (Figure 4a) from *Streptomyces sviceus* [93,94] are also possible. Such structural arrangement confers high stability to the peptides and hence they may be used as peptide scaffolds [91].

Cyclic bacteriocins are also modified peptides that contain a head-to-tail peptide bond linkage (involving condensation of the N- and C-termini) to generate the cyclic structure of the molecules [95]. All members possess α-helices of similar sizes that fold into a tertiary structure having a central pore surrounded by a compact globular bundle comparable to the saposin folds [96,97,98]. A good example is carnocyclin A (Figure 4b) from *Carnobacterium maltaromaticum* [99]. Furthermore, glycocins are a group of bacteriocins with one or more residues in the peptide chain linked to a carbohydrate moeity [1]. Sublancin 168 (Figure 4c) is an example of a glycocin from *Bacillus subtilis* with a *β*-S-linked glucose moiety [100,101]. Another group of bacteriocins called the sactipeptides contain sulphur-to-α-carbon linkages [1,90]. Extensive studies performed with subtilosin A (an example of this class) from *B. subtilis* [102] show that the carbon-sulfur linkages and hairpins are common structural elements [95,103,104]. Subtilosin A has three sulfur-to-α-carbon cross-linkages (Figure 4d) and demonstrate wide activity spectrum against a variety of bacterial strains [90,105]. Overall, the occurrence of these natural structural features, posttranscriptional modifications and unusual amino acids make the peptides more stable and effective as anti-infective agents.

Class I bacteriocins, also referred to as lantibiotics are ribosomally synthesized and comprise of non-proteinogenic amino acids and cross-linkages formed between dehydrated side chains of threonine/serine and the sulfhydryl group of cysteine. An example is NAI-107 (Figure 4e) from *Microbispora* sp. [106] where additional modifications such as hydroxylation of proline, halogenation of Trp and aminovinylcysteine also exist. Unnatural amino acid and thioether rings are prevalent in the different classes of lantibiotic [107,108] while hydroxylation, halogenation [57,106,109,110], and even therapeutically relevant moieties like *N*-glycosylation [111] are rarely present in a few examples. The posttranslational modifications in lantibiotics make them more resistant to protease degradation [112] and also accord them limited conformational freedom which confers target specificity [113]. The rigid conformational flexibilities facilitate high biological activities with very low minimal inhibitory concentrations (MICs) against wide variety of infections ranging from antimicrobial to antiallodynic effects [114].

Pediocin-like bacteriocins are heat-stable and are produced by a variety of LAB [115]. Their N-terminus has an overall positive charge, a disulfide bridge formed between two cysteine residues and a conserved YGNGVXC consensus motif (Figure 4f), which may be actively involved in target recognition and the killing process [115,116]. The disulfide bridge may play a stability role and not necessary directly involved in the killing process since its replacement by hydrophobic interaction did not abolish the activity of leucocin A [117]. An unmodified bacteriocin like the heat-labile Lactococcin 972 (Figure 4g) has two three-stranded antiparallel β-sheets that fold into a β-sandwich to confer stability [118].

The presence of unnatural amino acids in peptides revolutionize their overall activities against multidrug-resistant microbes with very low MIC [119,120]. Teixobactin (Figure 4h) is a non-bacteriocin peptide with interesting chemical constitution including four d-amino acids, enduracididine, an N-terminal methylphenylalanine and a thioesterase ring formed between Thr8 and Ile11 [121]. These features provide resilience and target-specific properties to most non-ribosomally synthesized peptides including vancomycin, bacitracin and valinomycin making them therapeutically more interesting.

It is worthy to note that altering the features that are responsible for activity or stability like hydrophobicity, α-helices, β-strands and unnatural amino acids may help to improve the therapeutic qualities of AMPs but may also produce negative effects on the desired functions. For instance, d-amino acids is important in enhancing biostability but its coexistence with l-amino acids tend to form special structural motifs that may be responsible for non-specificity and cytotoxicity [122]. However, altering these motifs in most cases result in drastic decrease in activity [123]. Meanwhile suboptimal structural arrangement of the peptide may take credit for such eventualities, it may be prudent to rethink on making these peptides more specific to make them safe for human consumption and ecofriendly to other organisms.

## 3. General and Specific Modes of Action of AMPs

Some reviews analyzed the different modes of action of AMPs and explained how bacteria may develop resistances against them by modifying their cell surfaces to sequester or repel the peptide, secreting proteases that inactivate them, producing transmembrane efflux pumps, forming biofilms and secreting molecules that may trap the AMP and inhibit their activities [9,124]. Although theoretical analyses of the pharmacodynamic differences between AMPs and conventional antibiotic with regards to their propensities to illicit resistance evolution show low probabilities for AMPs [125], extensive knowledge on the different molecular targets and their responses to AMPs’ interaction is required to fully understand and prevent these situations. Widespread opinions hold that most antibacterial peptides engage in membrane interactive mechanisms to inactivate their targets. However, this general believe has gradually faded away over the last two decades as numerous studies have identified different molecular targets (Table 1). Most AMPs exhibit conformational amphipathicity at the bacterial membrane interface to enhance electrostatic interactions with the negatively charged surface [126,127]. Irrespective of an AMP’s killing mechanism, the peptide may adopt a different conformation at the level of bilayer membrane to facilitate its activity. α-helical AMPs for example usually permeabilize the cytoplasmic membrane into subcellular compartments to interrupt essential cellular pathways [128].

**Figure 5:**
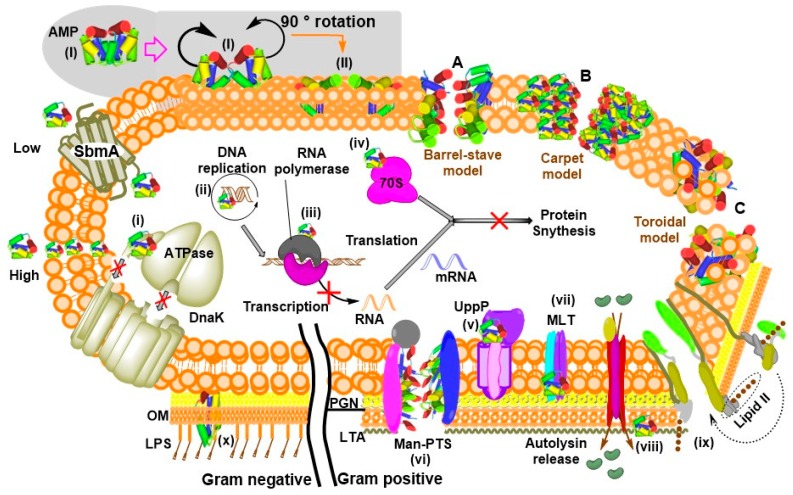
Membrane penetration mechanisms and mode of action of AMPs. Shaded region illustrates a structural model of the molecular interaction of an AMP with the phospholipid bilayer. **A**, Barrel-stave pore membrane disruption mechanism where the peptides line the hollow pore, and are oriented parallel to the phospholipid chains. **B**, Carpet mechanism where the peptides have a detergent-like effect on the membrane. **C,** Toroidal pore where the lumen of the pore constitutes a mixture of peptide and phospholipids resulting from perpendicular insertion of the peptides in the bilayer. Non-invasive mechanisms of AMPs include binding to: (**i**) DnaK, (**ii**) duplex DNA helices (**iii**) RNA polymerase, (**iv**) 70S ribosome, (**v**) undecaprenyl pyrophosphate phosphatase (UppP), (**vi**) mannose phosphotransferase system (Man-PTS), (**vii**) maltose transporter (MLT), (**viii**) Lipoteichoic acid (LTA), (**ix**) lipid II, inhibiting cell wall biosynthesis, and (**x**) LPS. The abbreviations PGN, peptidoglycan; OM, outer membrane.

A model by Sánchez-Barrena et al. describes the interactions between α-helical AMP and the lipid bilayer. As illustrated in Figure 5 (grey-shaded region), two protomeric units of the bacteriocin AS-48 interact via their hydrophobic and polar helices to produce two dimeric forms. The free-state dimeric form (I) targets the membrane and undergoes a transition from dimeric form (I) to the second dimeric form (II) at the membrane’s surface. Their non-polar regions are then buried into the hydrophobic core of the phospholipid bilayer while the polar helices interact with the polar heads [129]. Additionally, an earlier model described how cationic peptides cross the lipopolysaccharides (LPS) of Gram negative bacteria into the periplasm and subsequently engage the cytoplasmic membrane [130]. The simultaneous interaction and disruption of membrane bilayer may be achieved in different ways. Three well-established methods of how AMPs perform these actions include barrel-stave pore, carpet, and toroidal pore mechanisms [131,132] as illustrated in Figure 5. At low concentrations some AMPs, mainly PrAMPs, stereoselectively diffuse into the cell via the membrane protein SbmA to interfere with intracellular pathways by binding to molecular components such as DNA and RNA as well as cytoplasmic and membrane-bound proteins [70,132]. However, they may also nonspecifically invade the lipid bilayer at high concentrations (Figure 5).

Conceivably, the binding of AMPs to lipid bilayer are purposefully driven by the negatively charged surfaces of bacteria cells, contributed by the predominance of teichoic acids at the cell surface of Gram-positive [133] and the LPS of the outer membrane of Gram negative bacteria [134]. However, other opinions argue that sustained interaction at the outer membrane interface may constrain the effectiveness of AMP by decreasing the active concentration that finally arrives the cytoplasmic membrane where bactericidal activity is actually observed [135]. Furthermore, PrAMPs constitute the group of peptides that do not destroy the membranes, but engage in non-lytic inhibitory approaches against microbes via distinct molecular mechanisms involving cell penetration and intracellular localization [70,136,137,138]. PrAMPs may also interact and/or bind to LPS of Gram negative bacteria which initiates a conformational change whose rate is dependent on the structural properties of the compound [139].

Non-lytic peptides are obviously interesting to investigate further to identify additional properties that enable them to perform their functions. Some may also combine membrane disruption and cytoplasmic genomic DNA binding which further facilitates cell death [155]. The physiological responses of organisms when attacked by an AMP through various activity targets are described in Table 1, citing an overview of well-characterized examples.

## 4. Potential Applications and Accompanying Challenges

AMPs have a wide range of possible uses including therapeutic, template for creating new drugs and in medical devices; largely ascribed to their ability to elicit antimicrobial activity over a wide spectrum at low MICs. They bind specific targets, exhibit little endotoxic effects, act synergistically with conventional antibiotics, and display negligible tendencies to develop resistances [156]. Comprehensive reports describing therapeutic successes of AMPs with reference to the number of patents approved or applied for since 2003 were recently published [157,158]. These articles described the universal trends and approaches implemented in rational design of novel peptides and techniques used to decipher and evaluate possible routes of biological activity. Some loopholes such as susceptibility of the peptides to host protease inactivation and in vivo toxicity were also highlighted as associated factors that limit effective therapeutic applicability [158].

A variety of potential medical applications like the treatment of bacterial biofilms and associated complications [159], implementations in ectopic therapeutic operations, synergistic action in combination with conventional antibiotics against multidrug-resistant pathogens, and their use as building blocks for rational peptide drug design to overcome therapeutic challenges [160], were reviewed recently. Different AMP-containing formulation strategies have been considered to resolve the problem of chemical or biological inactivation of the drug molecule and also reduce adverse effects, thereby enhancing their potencies and safety [161]. Typical applicable examples in biomedical science like the potential use of cathelicidin-overexpressing bacteria to treat inflammatory bowel disease, taking advantage of its antifibrogenic effects in models of colitis, is possible [162]. In fact, endogenous AMP production has been suggested as an ideal approach for multiple disease treatment in humans and animals [127]. Furthermore, epithelial tissue repair, treatment of wound infections and promoting wound healing, that are chiefly inspired by the immune stimulatory properties of some AMPs are also attainable [163].

The generation of antimicrobial surfaces on medical devices is an interesting area for both researchers and medical practitioners especially for implant therapy. Different immobilization techniques exist [164] to create antimicrobial surfaces that are modified to leach out anti-infective agents onto the surface of the material or the surface itself is designed to possess bactericidal properties, thereby preventing contamination and reducing disease burdens from healthcare-associated infections [165,166]. Additionally, a plausible way to fight biofilm-related infections in clinical settings is to coat the abiotic surfaces of medical implantable devices with multifunctional AMPs that remain stable over prolonged periods, to supply anti-infective functions and/or prevent microbes from sticking to the device [156,167]. AMPs may be used in peptide surface coating, biosensors and detection, and nanoparticle-based drug delivery [9]; e.g., in microfluidic chip where the peptides were coated via cysteinyl interactions to a gold surface and used to specifically detect varied species of bacteria [168]. Low sample consumption in such experiments is an added advantage of surface covalent tethering of AMPs [32]. Interestingly, this strategy may also be used to identify possible mechanisms of action since only membrane disruptive peptides may easily elicit significant activity when immobilized. This may further reduce costs of pre-clinical evaluation with respect to amount of the product required for studies and hence encourage investments in this direction.

Tethering AMPs to solid surfaces via physical or chemical means may be compromised by the peptide’s disorientation, reduced flexibility, limited surface density of reactive groups, coupling conditions and steric hindrances, which may affect final biological activity [169,170]. Perhaps it is also difficult to obtain biomolecular structures of surface immobilized peptides. The physical immobilization technique may have some downsides that circumvent its extension to medical devices namely; short-term antibacterial effect and the leaching of antibacterial agents with potential environmental toxicity that may also encourage development of microbial resistances [171,172,173]. The same scenario could also be observed if AMPs were to be applied in disinfectants that are mostly used in hospital environments. It is conceivable that tethering AMPs to abiotic surfaces would require biostable, physically and chemically resistant molecules, which require huge capital and intellectual investments to achieve. Although realizing such a project may be an attractive venture opportunity, potential actors mostly feel apprehensive about the outcome.

## 5. The Paradox Surrounding Artificial AMPs and Semisynthetic Derivatives

Efforts to develop artificial antimicrobials have grown over time as scientists try to resolve the challenges of antimicrobial resistances. Although, this strategy allows for flexibility in designing active candidates, unaddressed concerns that may hinder progress in this direction seem likely to have a direct link to limited information that is available on their pharmacological properties. Poor intrinsic therapeutic qualities of AMPs such as biostability, solubility and bioavailability under physiological conditions, are amongst the strategic factors that restrict their clinical applications as drugs. Synthetic mimics are mostly inspired by the natural compounds’ abilities to kill multidrug-resistant pathogens at very high potency, while guaranteeing enhanced pharmacological properties like low MIC and low toxicity [119]. In general, both peptidic and non-peptidic synthetic variants have been inspired by the amphiphilicity of natural AMPs since numerous studies have identified this structural feature to be responsible for their functions. Many interesting molecules with significant functional properties have been produced such as AMPs with a hydrophobic center, flanked by two cationic regions [32] and K4R2-Nal2-S1 that possesses both antibacterial and anticancer activities [86].

The huge conformational flexibility of artificial peptides may cause them to be less specific thereby facilitating interactions with a wide range of targets and eventually producing side effects and unfavourable immunogenic responses [25]. As an alternative solution, semisynthetic modifications of naturally produced peptides may be used, which are efficiently produced in a bioprocess, but chemically or enzymatically modified afterwards [174]. Variants of deoxyactagardine B [175,176] and nisin [6] are such examples of how the clinical value of natural AMPs can be increased by the use of such combined strategies. Moreover, semi-synthesis is a greener process and produces higher yields within a shorter timeframe than total synthesis. Given that natural AMPs show little adverse effects and very low median effective dose, both in vitro and in vivo, in comparison to conventional antibiotics [45], it may be helpful to utilize their backbones as templates rather than creating entirely new synthetic peptides that may provoke unexpected side reactions. This can also be achieved by tackling modular approaches via the use of specific incorporated non-canonical amino acids into the peptide chain that warrant modifications via click chemistry [7,177]. Unnatural amino acids would expand the chemical reactivity space within a peptide and thus create room for enhancing biological activities as well as diversifying its applications [7,178].

Furthermore, the costs of developing a new peptide may be an argument for starting with a natural one which already has some kind of selectable biological activity. As an example, dendritic peptides like the G3KL described earlier are easier to produce and generally have a relatively simple structure [179]. Their stability enable them to be used in several applications including antimicrobial and tumor-targeting functions [180]. However, care must be taken when considering them as drug candidates since an example like the G3KL possesses structural features that are analogous to antibodies, which consequently may provoke undesired immunogenic responses. The fact that they can self-assemble into helical pores [181] may also increase their toxicity to higher organisms preceding insertion of the hydrophobic regions into the lipid bilayer membrane. Attention must be drawn to the fact that this is just one of many structural features that may result from an artificial peptide design. Even more worrying is also the fact that some of the features (e.g., PPII helix) allow interaction and membrane translocation of the molecules. Paradoxically, there exists a plethora of research supporting the design and use of artificial peptides as alternative drug candidates. We are of the view that utilizing natural AMPs as backbones in semisynthetic processes is an effective way to design novel compounds and to develop lower-cost production processes that are amenable to economically feasible industrial applications. Moreover, having a natural backbone is advantageous in that the properties of the new molecule may not deviate very much from the original one, and therefore its safety limits would not be narrowed.

## 6. Controlling the Unspecificity of AMPs

This section focusses on possible structural and molecular bases of unspecificity since details of the kind of conformational transitions that a peptide adopts at the bilayer membrane are subjects still under investigation to decipher the molecular mechanisms of toxicity. Analyzing the physicochemical parameters that influence attraction of a peptide to the cytoplasmic membranes of both bacterial and eukaryotic cells, may be very helpful in therapeutic considerations once a correlation with a biological assays is established. Some AMPs that have significant bactericidal activity profiles also display higher levels of hemolytic activities like the recent cases of EPrAMP1 and EeCentrocins [37,57]. Methods such as adding d- and fluorinated amino acids into the core peptides to restrict targeting of host cells have been discussed earlier [182]. However, substituting residues for d-amino acid or proline in respective AMPs considerably reduced their amphiphilicity and/or helicity, decreased binding affinities to cell membranes as well as bactericidal activity [183,184]. A similar approach was used earlier to reduce cytotoxicity of AMPs marked by eukaryotic cell membrane damage [185]. Therefore a compromise must be anticipated between the desired biological effect and the overall applicability of the final product.

The structural motifs of the non-lytic PrAMP may be interesting for rational design or modifications of already existing peptides to improve their activities and reduce toxicity to eukaryotic cells. A comparison between the cytotoxic activities of Pro, Lys and Arg homopeptides with increasing peptide chain lengths showed a trend with proline consistently displaying lower cytotoxicity [72]. Nonetheless, little is known about the interactions of specific groups or the PPII helical conformation of PrAMPs with eukaryotic cells. A recent publication described a couple of methods that may be used to investigate bacterial membrane permeabilization and localization of PrAMPs in the cytoplasm where they interfere with cellular functions [186]. The review postulated a variety of means to generate data that would eventually enhance the design of potent antimicrobial agents, however, the molecular description of how the peptides translocate across the cell membrane that could perhaps have strengthened these suggestions are scarcely available in current literature. Nevertheless, a recent study by Tossi’s group showed that human LL-37 forms covalently linked hexamers at higher concentrations and also demonstrated a strong correlation between the type of oligomerization and antimicrobial activity, as well as nonspecific disruption of target [187]. Prior to this work, already Johansson et al. (1998) had established a model that described the dependence of the formation of α-helices and the concentration of a peptide to the following oligomerization. The authors recognized the hydrophobic effect of the oligomers as the driving force behind the conformational rearrangement [188].

Although it was suggested that disrupting helix formation may be a conceivable means of mitigating cytotoxicity of AMPs [189,190], it would not be logical to proceed as such since the approach also decreased the anti-infective properties of the compounds. Both cytotoxicity and antimicrobial activity of an AMP are equally important during therapeutic evaluation and therefore it is necessary to first consider the physiological environment where it will act and how much potency is desired to tune the peptide to achieve maximum clinical effects. Moreover, the presence of α-helices and β-strands in a peptide, combined with increased cationicity and amphipathicity leads to improved antibacterial effects. On the contrast, reducing its charge or hydrophobicity and amphipathicity results in simultaneous decrease in potency and cytotoxicity [28,128,191,192]. Schmidtchen and colleagues discussed how hydrophobic modifications of AMPs may provide alternative means of addressing these issues by supplying information on how to correlate physicochemical observations to biological realities [193]. Although it is unclear why an AMP like EeCentrocin 2 is highly haemolytic, engineering the peptide to reduce its hydrophobicity at the helix hydrophobic face may significantly decrease its cytotoxicity, though at the expense of antimicrobial activity [182].

The positions and types of hydrophobic residues distribution play a key role in the antimicrobial activity of an AMP and have therefore enabled the rational design of a few compounds with potent activities [184,194]. For example, it was reported that the addition of β-naphthylalanine to the C- or N-terminus of an AMP induces higher cytotoxicity [195], while incorporating positive charged residues like lysine and arginine to the N- and/or C-terminus reduced toxicity to human red blood cells and human fibroblasts [86,196]. Interestingly, incorporating a bulkier hydrophobic group into an inactive chymotrypsin-resistant human LL-37 peptide revived its anti-MRSA properties and also played an additional role by recruiting monocytes to the infection site [197].

Finally, AMPs function synergistically with conventional antibiotics [198,199] and as such, coupling the antibiotic molecule to the peptide via chemical means may help to improve stability and limit likelihood of microbial resistance by (i) offering biological and physicochemical resistance to the peptide without altering the peptide chain and (ii) multiple killing mechanisms by the conjugated compounds which may allow for lower MICs incapable of eliciting toxicity to host cells or development of resistances by the targeted organisms. This type of modification has already been reported where a diaminoalkane chain and a 3,5-dichlorobenzylamine group were coupled to the C-terminus of deoxyactagardine B to produce NVB302 and NVB333 [175,176], both having very good pharmacokinetic/pharmacodyanamic profiles [27]. Nevertheless, the complexity and cost of producing each component, as well as deciding at which position to incorporate the drug molecule to the peptide chain are some of the questions that extensive research in this area may help to resolve.

## 7. Molecular Premise for Evaluating the Ecotoxicity of AMPs

Generally, AMPs especially those of class I bacteriocins, as well as unnatural amino acids containing members have complicated structures that are not easily degraded via natural enzymatic processes. They also have high resistance to heat and chemical inactivation which may prevent degradation of the compounds both in vitro and in vivo. Whether these properties represent potential environmental risk factors remains an open question. We try to address this question by discussing some molecular interactions that draw attention towards the topic. First and foremost, AMPs may leach out from AMP-coated devices, manuring and in aquaculture to the surrounding environments. If consumed in food and animal feed (as preservatives) or as therapeutic drugs, they may be released into the wastewater effluent from home reservoirs.

Proposing a methodology to evaluate cellular targets for AMPs and the mechanisms of their action is technically challenging and risky because of the complexity of their killing mechanism which may involve more than one target, and thus require sophisticated methodological approaches. Nowadays, several methods have been developed for that purpose ranging from phenotype and metabolite profiling, to microscopic and biophysical studies using artificial membranes, different types of hemolytic assays, endotoxin-release assays etc. Since optimal assay conditions may vary depending on the expected outcomes, integrative approaches that incorporate short-term and long-term perturbations by a compound on the environment would be effective in toxicity screening. However, before evaluating toxic mechanisms like genotoxicity, oxidative stress responses, ATP leakage, nucleic acids binding etc., demographic endpoints like organism’s survival, growth inhibition, motility, population changes and reproduction must have shown clear indications [200] that ascertain the ecotoxicity risk of an AMP. A summary of standard tests that are routinely used to assess the environmental risks of chemicals, including details of environmental species, exposure and test endpoints are described elsewhere [200,201]. Meanwhile present ecotoxicological evaluation focus predominantly on nanoparticles and other industrial chemicals that are occasionally leached to the environment, attention has rarely been given to AMPs despite structural similarities of some members to unfriendly toxins [36,37,38]. We discuss here some aspects of AMPs’ killing mechanisms that directly or indirectly interrupt survival, growth or reproduction, and examine how these events may factor into potential environmental risks and how they may be assessed.

### 7.1. Cell Wall

Like vancomycin and isoniazid, AMPs such as polybia-MPI, G3KL, EeCentrocin 1 and 2 and indolicidin kill their targets by disrupting the cell wall [57,84,202,203]. Cellulosic or chitinic multilayered cell walls are common in fungi, algae and higher plants while bacteria contain Lipoteichoic acid (LTA) and lipopolysaccharides (LPS). These structures constitute defensive barriers that when compromised may result in deleterious consequences. Although unspecific disruption of cell wall may be considered as a potential threat, LPS distinguish prokaryotic cell envelopes from those of eukaryotes by having lower anionic charge and high cholesterol levels [204]. Since some AMPs elicit antimicrobial activities via LPS [12,33,137,139,151,152] and because of its ubiquitous presence in microorganisms, the LPS layer may be a specific target to treat infections. An acute toxicity evaluation of environmentally friendly microbes that possess this unique feature may be performed using the luminescent bacterium *Vibrio fischeri*. Gram staining, SR2200 dye [205] and Lugol’s solution [206] may be used in conjunction with transmission and/or scanning electron microscopy to investigate the status of the cell wall.

### 7.2. Lipid Bilayer Membrane and Pore Formation

The size, cationic charge and amphipathic nature allow interactions and subsequent insertion of AMPs into the lipid bilayer membrane to form pores via several mechanisms described as ‘carpet’, ‘barrel-stave’ or ‘toroidal-pore’ [132]. Pore-forming AMPs constitute an integral part of the pathogenic microbial virulence arsenal [207]. Epithelial barriers are usually compromised by pore-forming toxins secreted by proliferating bacterial pathogens that help to modulate or disrupt the host immune system, producing cellular responses such as changes in ion concentrations and membrane repairs [208]. Reports show that cytotoxicity to mammalian cells is observed at elevated concentrations of AMP, plausibly via apoptotic mechanisms of cell death [209]. Although AMPs can perform their desired function at suboptimal concentrations, it is necessary to be able to evaluate their effects in a broad range since the peptide may accumulate to build concentrations that exceed safety limits. For example EeCentrocin 2 has an unexplained high hemolytic activity [57], which may apply to other AMPs as well. Their abilities to nonspecifically damage eukaryotic cell membranes drives the need for ecotoxicity evaluation. This is because indiscriminate disruption of the cell membrane could constitute an adverse condition that limit the proliferation of non-target organisms. Hemolytic assays such as the lactate dehydrogenase activity assay may be used to assess hemolysis while methods like the anilinonaphthalene-8-sulfonic acid uptake [210] or the dye leakage assay [211] may be used to measure concentration-dependent membrane disruption.

### 7.3. Nucleic Acid Binding or Damage

Stereoselective diffusion across the plasma membrane into the cell is a common phenomenon exhibited by non-lytic peptides like the PrAMPs [70,132]. The hypotheses that translocated AMPs can inhibit enzymatic activity, nucleic acids or protein syntheses are supported by a few studies [11,145,146,153,154]. Interruption of intracellular pathways may in fact be a bigger problem since it cannot be easily detected. CM15 is an example of AMP that induces oxidative stress in *Escherichia coli* [212]. Oxidative stress produces reactive oxygen species that may damage DNA resulting in slow development and low production yields of plants [213] and death of fungal cells [214]. The possibilities that these events might have only long-term effects on the affected organisms should be considered with extreme caution. Therefore, it is necessary to evaluate the extent to which AMPs interact with nucleic acids so that their effects can be better controlled. In plants, oxidative stress responses and other physiological changes in photosynthetic pigment, enzymes activity and lipid peroxidation may be measured as described elsewhere [215,216,217]. Techniques such as CellROX Green or Amplex Red oxidation assays [214] may be used to evaluate similar situations in smaller microbes. AMPs may bind cytoplasmic genomic DNA to facilitate cell death [155]. The Comet assay has proven to be a sensitive, rapid and reliable tool for assessing genotoxicity [218,219,220]. It is also important to investigate the specific molecular interactions that facilitate these events in order to establish methods to control them.

### 7.4. Membrane Transporters and Receptors

So far, MLT, Man-PTS, UppP and MBM have been identified as targets for AMPs activity. These transporters/receptors are important cellular components that enable their smooth functioning. Their interactions with AMPs [for example Garvicin ML with MLT [140] or Lactococcin G with UppP [10]] may be suicidal to the cells since inhibition of the membrane-located receptor may disrupt transportation of sugar or cell wall synthesis. Furthermore, the interaction of LsbB with Membrane-bound metallopeptidase [13] can cause a lot of complication due to accumulation of a misfolded proteins (that would have perhaps been degraded by the MBM) leading to septic shock. We believe that membrane transporters may also function as a docking site for the peptide or that they may be permeable to the molecules, allowing them to cross the membrane and attack other yet to be identified intracellular targets. The identification of the UppP and the metallopeptidase receptors, open new avenues to suggest that the heterogeneous structures of lanthipeptides for example, may have different targets and different killing mechanisms that are not yet known [221].

Sugar transport across the cell membrane may be assessed using rudimentary approaches that measure sugars uptake by cells. Flow cytometry or fluorimetry or liquid scintillation counting may be used in conjunction with a fluorescent sugar analogue like the 2-[*N*-(7-nitrobenz-2-oxa-1,3-diazol-4-yl) amino]-2-deoxy-d-glucose (2-NBDG) to measure sugar transport across the cell membrane.

The ToxTracker assay [222] which has been extended to identify differences in cellular responses to DNA damage [223] may be used to assess protein misfolding and oxidative stress.

### 7.5. Chaperones

Apidaecin, pyrrhocoricin and drosocin are PrAMPs that displayed defined interactions with *E. coli* DnaK [224]. However, inactive pyrrhocoricin analogue together with cecropin A or magainin 2 which are membrane-active AMPs did not show any inhibition of this macromolecule [147], indicating structural specificity. The binding of the peptides to this chaperone inhibits its ATPase activity resulting in accumulation of non-functional misfolded proteins [225]. Since all living organisms depend the smooth functioning of the heat shock proteins (chaperones), it makes sense to evaluate the potential effects of AMPs against different environmental species. The ToxTracker assay mentioned above may be used to sense protein misfolding that may result from likely inactivation of the chaperone system by an AMP. However, extensive sequence variation at various domains of the macromolecules in different species makes it possible to design a peptide that would specifically inhibit a target organism, eliminating cross-reactivity that may trigger adverse reactions to the host or facilitate development of resistances.

### 7.6. RNA polymerase and 70S Ribosomes

Bac7_(1–35)_, and other insect-derived PrAMPs were shown to bind or interact with 70S ribosomes [143,144] while Microcin J25 and Capistruin interacted RNA polymerase [145,146]. In all the reported cases, gene transcription or translation was blocked in the presence of peptide. Structural and biochemical data show that interaction of PrAMPs with the ribosome occur within the exit tunnel where the peptide binds with a reverse orientation and prevent nascent polypeptide chain from entering the elongation phase of translation [79]. Inhibition of transcription and translation can directly affect survival, growth or reproduction. The anti-infective agents acting as inhibitors of these processes obliges further investigations to identify if they can in fact cause significant damages to the ecosystem, but again these macromolecular complexes have dissimilar structural features which serve as a plus in target-specific peptidomimetic drug design.

## 8. Conclusions

Several screening methods have enabled isolation and characterization of novel antimicrobial peptides both from natural and synthetic sources. Special features are required in the peptides for them to perform the desired functions, provide resilience and target specificities that make them therapeutically interesting. Although the market situation for AMPs like Bacitracin, Polymyxin and Fuzeon^®^ have encouraging records, there is no doubt that AMPs face a lot of developmental challenges ranging from production to clinical development. These obstacles are conflicting with the numerous academic publications that appear every year to argue the potentials of these biomolecules as alternative drug targets. Perhaps better understanding of the mode of action, target molecules or complex, and mechanism of host protection may further improve the current situation and reduce time and capital investments which may be frustrating at best in the event of an unsuccessful venture. The solution to these challenges must be multifaceted in nature. This may include, amongst others, utilizing natural AMPs as backbones in rational semisynthetic desins to improve their therapeutic qualities and engineering the peptides to reduce toxicity to host cells, or increase their activities.

Membrane interactive models like the toroidal pore formation, carpet and barrel-stave pore have allowed the possibilities to evaluate how AMPs affect bacteria based on their conformational flexibilities and nature of the lipids in the bilayer and thus, understanding the molecular interconnectivities of the peptide-target interactions may greatly facilitate the creation of new molecules using the current ones as templates. Analysing the molecular bases of targeting intracellular components is also important to identify the types and nature of interactions involved, which may provide information on how to develop peptides with superior potencies and the propensity to escape bacterial resistances. Meanwhile suboptimal structural arrangement in the peptides may result in drastic decrease in activity, it may be useful to design peptides that are more target-specific to make them safe for human consumption and ecofriendly to other organisms.

Nevertheless, natural AMPs show high potency against pathogenic microbes, targeting multiple cellular macromolecular structures whose constituent building blocks or conformational dynamics vary from species to species and therefore, resistance development is rare. As such, even if there is wide exposure to the environment like in the hospital settings, AMPs may instead contribute to prevent healthcare-associated infections in hospital settings and food-borne illnesses so long as environmentally friendly species are not targeted.

## Figures and Tables

**Table 1:** Characterized AMP targets and their killing mechanisms.

Target Molecule	Killing Method	Examples	Reference
MLT	Efflux of intracellular and/or influx of extracellular solutes	Garvicin ML	[140]
Man-PTS	Efflux of intracellular and/or influx of extracellular solutes	Pediocin-like bacteriocins, Lactococcin A, microcin E492	[73,141]
UppP	Disrupts cell-wall synthesis	Lactococcin G and Enterocin 1071	[10]
MBM	Prevent proteolytic breakdown of a misfolded protein	LsbB	[13,142]
70S ribosome	Inhibit protein synthesis	Bac7_(1–35)_, insect-derived PrAMPs	[143,144]
RPol	Inhibit transcription by obstructing RNA polymerase activity	Microcin J25, Capistruin	[145,146]
DnaK	Inhibit ATPase activity	Pyrrhocoricin, Bac7 (PrAMPs)	[137,147,148]
LTA	Release of autolysin	RTD2 and Pep5	[149]
Lipid II	Inhibit cell wall biosynthesis, pore formation	nisin	[150]
LPS	Restrict LPS binding to CD14+ cells and hence prevent fatal septic shock syndrome Interact with AMP and enable folding	Human cathelicidin LL-37 Indolicidin, magainin 2, cecropin A, esculentin-derived AMP, pyrrhocoricin	[12,33,137,139,151,152]
DNA/RNA	Inhibiting DNA replication	Indolicidin, Buforin II, tachyplesin, RR4	[11,153,154]

Abbreviations: MBM, Membrane-bound metallopeptidase; RPol, RNA polymerase.
